# Contrasting Profiles of Problematic Internet Use among Tunisian University Students: Computer Science versus Law Majors

**DOI:** 10.1192/j.eurpsy.2026.10841

**Published:** 2026-07-31

**Authors:** M. Mouldi, M. Khamassi, H. Ghabi, M. Karoui, H. Nefzi, R. Kamoun, F. Ellouze

**Affiliations:** Department of Psychiatry G, Razi Hospital, Manouba, Tunisia

## Abstract

**Introduction:**

Internet addiction represents an emerging public health concern, particularly among university students. Academic demands and disciplinary contexts may shape both the patterns of Internet use and the susceptibility to problematic behaviors. This study compares two distinct student populations: computer science majors, whose studies require extensive online engagement, and law students, whose digital interaction is generally less integral to their curriculum.

**Objectives:**

This study aimed to compare the prevalence and characteristics of problematic Internet use between Tunisian university students majoring in computer science and those majoring in law.

**Methods:**

A cross-sectional study was conducted among students enrolled at the National Institute of Applied Sciences and Technology (INSAT) and the Faculty of Legal, Political and Social Sciences of Tunis (FSJPST). Participants completed a self-administered questionnaire assessing sociodemographic characteristics, time spent online, motivations for Internet use, and scores on the Internet Addiction Test (IAT). Data were analyzed using descriptive statistics, χ^2^ test, Fisher’s exact test, and ANOVA.

**Results:**

A total of 113 students were included. The mean IAT score was 41.8 ± 17.7. Overall, 45.1% of participants exhibited mild addiction, 27.4% moderate addiction, and 2.7% severe addiction, while 24.8% showed no significant addiction. Computer science students demonstrated a higher proportion of moderate-to-severe addiction (31.2%) compared with law students (less than 20%), although the difference did not reach statistical significance (p = 0.136). Recreational use was the most frequently reported motivation (67.3%) and was significantly associated with addiction severity (p = 0.024). A self-perception of excessive use (reported by 66% of students) correlated with higher severity (p = 0.040), and attempts to reduce usage (52.2%) were more common among those with moderate-to-severe addiction (p < 0.001). No significant associations were observed with gender, age, place of residence, or daily time spent online.

**Conclusions:**

Problematic Internet use was highly prevalent among the studied population, with a greater, though not statistically significant, tendency among computer science students. Addiction severity was primarily associated with motivational (recreational use) and perceptual (self-recognized excessive use) factors. These findings highlight the need for targeted awareness and preventive interventions within academic institutions, tailored to the specific risk profiles of different student disciplines.

**Disclosure of Interest:**

None Declared

